# The Effect of Acute Physical Exercise on NK-Cell Cytolytic Activity: A Systematic Review and Meta-Analysis

**DOI:** 10.1007/s40279-020-01402-9

**Published:** 2021-01-04

**Authors:** Christopher Rumpf, Sebastian Proschinger, Alexander Schenk, Wilhelm Bloch, Amit Lampit, Florian Javelle, Philipp Zimmer

**Affiliations:** 1 Faculty of Medicine, University Hospital Cologne, University of Cologne, Cologne, Germany; 2 Clinical Exercise-Neuro-Immunology Group, Department of Molecular and Cellular Sport Medicine, German Sport University Cologne, Cologne, Germany; 3 Institute of Sport and Sport Science, TU Dortmund University, August-Schmidt-Strasse 1, 44227 Dortmund, Germany; 4 Department of Psychiatry, The University of Melbourne, Melbourne, Australia; 5 Department of Neurology, Charité-Universitätsmedizin Berlin, Berlin, Germany

## Abstract

**Background:**

Data on changes in natural killer cell cytolytic activity (NKCA) in response to acute physical exercise are contradictory.

**Objective:**

The aim of this systematic review, meta-analysis and meta-regression is to (1) examine the effect of acute physical exercise on NKCA, (2) shed more light on the moderating factors, and (3) test the assumption of NKCA suppression subsequent to performing sports.

**Methods:**

Two comparisons of NKCA were performed: (1) pre- versus post-exercise and (2) pre-exercise versus recovery. Data were acquired through a systematic search of MEDLINE (via PubMed), Scopus, and SportDiscus. Studies were eligible for inclusion if the effect of acute physical exercise was assessed including a passive control group and reporting NKCA prior to and immediately after the trial, and during the first 2 h of recovery. To better explain between-study heterogeneity, a moderator analysis was conducted.

**Results:**

Pooled estimate from 12 studies reporting 18 effect sizes show that NKCA is largely elevated by acute physical exercise (Hedges’ *g* = 1.02, 95% CI 0.59–1.46, *p* < 0.01). Meta-regressions reveal that this effect is larger for endurance versus resistance exercise and increases with the intensity of exercise (both *p* < 0.01), whereas the blood material used in the assay (*p* = 0.71), and the quantitative change in NK-cell count (*R*^2^ = 0%, *p* = 0.55) do not play a significant role. Physical exercise does not affect the level of NKCA after the recovery period (*g* = 0.06, 95% CI − 0.37 to 0.50, *p* < 0.76).

**Conclusions:**

This work provides solid evidence for elevated NKCA through performing sports which returns to baseline during the first 1–2 h of recovery, but not below the pre-exercise values providing counterevidence to the assumption of temporarily reduced NKCA. Remarkably, the functional change in NKCA exists independently from the quantitative change in NK-cell count.

PROSPERO registration number: CRD42020134257.

**Supplementary Information:**

The online version contains supplementary material available at 10.1007/s40279-020-01402-9.

## Key Points

**Table:** 

Acute physical exercise has a large and positive effect on the level of NK-cell cytolytic activity in the peripheral blood (*g* = 1.02, 95% CI 0.59–1.46, *p* < 0.01).
In the first hours after performing sports, NKCA values return to baseline, but do not drop below the pre-exercise values (*g* = 0.06, 95% CI − 0.37 to 0.50, *p* < 0.76).
The data suggest the counterintuitive conclusion that the level of NKCA is not significantly altered by the changing numbers of NK-cells (*k* = 11, beta = 0.123, *R*^2^ = 0%, *p* = 0.55).

## Introduction

As part of the cellular innate immune system, Natural Killer cells (NK-cells) play a crucial role in the host defence against virus infected and neoplastic cells. NK-cells are characterized by a detection and killing of target cells without prior immunization and therefore build a first line of defence. The cytotoxic function of NK-cells is described by their cytolytic activity (NKCA), consisting of two major mechanisms. On one hand, NK-cells could provide cytotoxicity by secretion of cytolytic granules, and on the other hand by the induction of cell death receptors [1]. Cytolytic granules include effector proteins such as perforin and granzymes. Perforin is a pore-forming protein. In detail, it builds a membrane-attack-complex on target cells, causing pores, which in turn induce osmotic lysis [2]. Granzymes are serine-proteases inducing apoptosis and mediate cell death [1]. Cell death receptors are expressed on the cell surface and consequence in apoptosis upon ligand binding. NK-cells express the ligand of the cell death receptor FAS (FASL) and the TNF-related apoptosis-inducing ligand (TRAIL) on their surface [3].

Regulation of NK-cell function is achieved by a keen balance of signals of activating and inhibiting receptors that provide a discrimination between healthy and diseased cells. According to their function, NK-cells can be divided into two major subsets based on their surface expression of the cluster molecule CD56 [4]. On one hand, NK-cells with low levels of CD56 (CD56^dim^) that are highly cytotoxic and on the other hand, NK-cells with high levels of CD56 (CD56^bright^) that secrete cytokines and are regulatory.

Research in the field of exercise immunology has proposed both, a mobilization and redistribution as well as alterations in the function of these cells during and after exercise [5]. Today, it is well accepted that NK-cell mobilization during and shortly after cessation of exercise is mainly driven by epinephrine [6]. In contrast, less is known about NK-cell distribution to different tissues.

Besides NK-cell mobilization and redistribution, exercise was supposed to change NK-cell function in a dose- and time dependent manner. In detail, NK-cell cytolytic activity (NKCA) has been described to increase during various types of acute physical exercise and to decrease after sustained moderate and prolonged exercise [7]. It was speculated that the latter, contributes to an enhanced risk for upper respiratory infections since NK cells serve as “first line defence” against pathogens in the respiratory tract [8]. However, evidence for exercise-induced immune suppression is sparse and opposing opinions are also well accepted by the scientific community [9]. In contrast to the profound and explicit knowledge on exercise induced NK-cell mobilization, data and opinions on changes in NKCA are contradictory.

This inconsistency may be explained by numerous factors, such as strongly varying exercise paradigms (type, intensity, duration), measurement time points and populations as well as using different laboratory techniques (flow cytometry, cytochrome release assay, calcein assays, etc.) and biomaterials (whole blood, peripheral blood mononuclear cells or isolated NK-cells; for a review read Zimmer et al. [5]).

Against the backdrop of the increasing relevance of exercise in preventive and rehabilitative settings, it is of interest to quantify the effect of acute and mid-term (recovery phase) exercise-induced alterations in NKCA. Furthermore, this study attempts to identify potential technical and physiological moderators since these have been neglected in a previous meta-analysis [7].

Another critical issue relates to the confounding effect of NK-cell mobilization in response to physical exercise, and thus, many studies cannot reliably differentiate between the functional change in cytotoxicity and the quantitative change in NK-cell numbers. To sum it up, moderating factors on the individual, exercise, and methodological level are indispensable for drawing reliable conclusions on NKCA in response to physical exercise. Given the lack of coherent knowledge this research attempts to reach three major goals: (a) thoroughly examine the immediate effect of acute physical exercise on NKCA, (2) shed more light on the moderating factors, and (3) test the assumption of temporarily reduced NKCA suppression subsequent to performing sports.

## Materials and Methods

This study was conducted in accordance with the Preferred Reporting Items for Systematic Reviews and Meta-Analyses (PRISMA) [10]. All criteria presented in the PRISMA checklist were satisfied. The protocol was pre-registered on PROSPERO (registration number CRD42020134257).

### Search Strategy

The literature search was conducted using MEDLINE (via PubMed), Scopus, and SportDiscus. Since research on NKCA alterations in response to sports started in the 1980s [5], the literature search was performed over a period of 40 years (January 1980 to December 2019). The search strategy involved Medical Subject Headings (MeSH) and text words combined through Boolean operators (‘‘AND’’, ‘‘OR’’). The search string included common synonyms for the concepts of (1) physical exercise, (2) natural killer cells, and (3) NKCA: (exercise OR “physical activity” OR sport OR training OR cycling OR walking OR swimming OR running) AND (“natural killer cells” OR “natural killer cell” OR “NK-cells” OR “NK-cell”) AND (cytotoxicity OR cytotoxic OR “natural killer cell cytotoxic activity” OR “cytolytic activity” OR NKCA OR “cell activity” OR “NK cell function”). The scope of the search was restricted to peer-reviewed articles published in English. After deduplication and screening for the selection criteria, 74 full-text articles were independently assessed by 2 reviewers (SP and CR). Finally, 12 studies were included in the systematic review and meta-analyses.

### Selection Criteria

Participants: Studies using adult healthy human participants were included in the analysis.

Intervention: All studies comprising a single bout of physical exercise (e.g., running, cycling, resistance training) were eligible for analysis. Any exercise trials including other interventions (e.g., heat exposure) [11] or supplementations (e.g., naltrexone administration) [12] associated to exercise were excluded from the analysis.

#### Comparison

Only studies including passive controls (e.g., resting, sitting) were included in the review.

#### Outcomes

Change in NKCA from pre- to post-exercise (i.e., immediately after) between the experimental and control groups were the primary interest of the analysis. Change in NKCA from pre-exercise to post-recovery, between the experimental and control groups were the secondary interest in the analysis. Studies with unclear data or incompatible outcome measures (e.g., gene expression, dim-bright ratio) were excluded from the analysis.

#### Study design

Only peer-reviewed randomized or non-randomized controlled trials were included in the analysis. Case studies, correlation studies, editorials, reviews, observational research, conference abstracts as well as studies with secondary analyses from subgroups of already existing and published data were excluded from the review.

### Data extraction

To accurately extract numerical data from plots if raw data were not reported, the software tool WebPlotDigitizer 4.2 was used. Subject characteristics such as gender and age, study design, exercise paradigm, blood sampling interval, type of NKCA assay, mean values and standard deviations (or standard error of the mean) of NKCA for each group in percent or Lytic Units were extracted. If publications did not report the required data, the corresponding author was contacted to retrieve the missing data.

### Quality assessment

Internal validity and risk of bias within included studies were assessed with the Physiotherapy Evidence Database scale (PEDro) [13]. The PEDro scale contains 11 items covering eligibility criteria, random allocation, concealment of allocation, comparability of groups at baseline, blinding of patients, therapists and assessors, analysis by intention to treat, between-group statistical comparisons and point measures and variability data. As exercise training interventions do not provide an opportunity to fully blind participants and therapists, items 5 and 6 from the PEDro scale were excluded from the grading process, meaning that the maximum PEDro score was nine.

Each criterion on the PEDro scale were rated as “yes” (indicating that the criterion has been met) or “no” (indicating that the criterion has not been met). The quality of each study was independently assessed by the same two reviewers (SP and CR) with an intraclass inter-rater correlation coefficient of 92%. In case of disagreement between the two reviewers, a third reviewer (AS or PZ) was consulted. ESM (electronic supplementary material) table A presents the total PEDro score for the included studies.

### Statistical analysis

All analyses were performed with the statistical computing software R using the packages metaSEM and metafor. Two separate meta-analyses were performed: (1) post-exercise NKCA versus pre-exercise NKCA for the exercise group versus the control group, and (2) recovery NKCA versus pre-exercise NKCA for the exercise group versus the control group. As NKCA was expressed in different and nonconvertible units across studies (i.e., cytotoxicity in percent versus Lytic Units), change in NKCA values were converted to standardized mean differences, calculated as Hedges’ *g* to account for small study sample sizes.

A positive Hedges’ *g* denotes an increase of NKCA in the exercise group over the control. By convention, a Hedges’ *g* value of 0.2 is considered a small effect size, 0.5 is considered moderate and 0.8 is considered large [14, 15]. Outcomes across studies were pooled using a random-effects model. The prediction interval was also computed to consider the potential effect of physical exercise when it is applied within an individual study setting, as this may be different from the average effect [16].

Between-study heterogeneity was measured using *τ*^2^ (variance of true effects) and further assessed using the *I*^2^ statistic which assesses the proportion of between-study variance over total observed variance [17]. An *I*^2^ value of 75% was considered large, of 50% moderate, and of 25% low. Potential small study effect (and potential publication bias) was assessed by visually inspecting funnel plots of standardized mean difference against standard error and using Egger’s test [18, 19]. If evidence for asymmetry was found (*p* < 0.1 on the Egger’s test), the Duval and Tweedie trim and fill method was used to quantify the magnitude of small study effect. Cook’s distance was used to determine outliers across studies [20].

To detect moderator effects, subgroup analyses were applied to the categorical variables of interest (i.e., gender, age, type of exercise, level of intensity, type of blood sample), while meta-regression was used to analyse the moderating effect of the continuous variable NK-cell count [21]. For this purpose, an NK-cell Hedges’ *g* effect size was emitted from change in NK-cell count from pre- to post-exercise (i.e., immediately after) between the experimental and control groups. The predictive value of continuous moderators was evaluated by goodness of fit (*R*^2^) and was significant at the *p* = 0.05 level.

## Results

### Overview of the systematic review

**Fig. 1:**
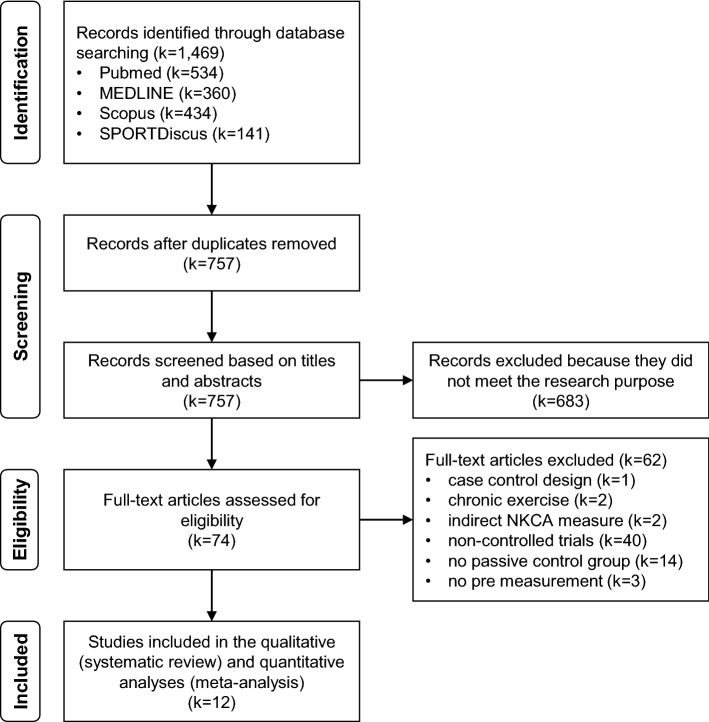
Literature search and results

The search strategy led to 1469 results in the literature databases. After removing duplicates, 757 studies were screened against the eligibility criteria. By reviewing the titles and abstracts, 683 studies were excluded and 74 were assessed for eligibility. After applying the selection criteria, 62 studies were excluded based on the full-text version. Finally, 12 studies were included into the analysis. A study selection flow chart is provided in Fig. 1.

The 12 studies included in the systematic review were published between 1995 and 2006. Sample sizes varied between 8 and 64 participants. Four of the studies tested only female participants [22–25], seven studies tested only males [11, 12, 26–30], and one study included both genders [31]. The participants’ mean age ranged from 21.6 to 72.8 years with ten studies testing young participants (< 40 years) [11, 12, 22, 25–31], and two studies testing for effects in older adults (> 65 years) [23, 24]. Ten studies used a randomized approach to assign participants into exercise or passive (i.e., sitting or resting) control groups [11, 12, 22, 23, 25–31].

Endurance exercise was used in eight studies [11, 12, 22, 25, 27–29, 31], three studies tested the effect of resistance training [23, 24, 30], and one study used both forms of exercise in a crossover design [26]. Following the terminology used by Norton et al. [32], exercise intensity was light in two [25, 27], moderate in six [11, 22, 26, 30], vigorous in seven [12, 25, 28, 29], and high in three trials [11, 23, 24]. The duration of the exercise trials varied between 5 and 120 min. As indicated in Table 1, blood samples were taken prior to and immediately after the exercise, whereas the timing of the final blood draw differed considerably due varying recovery periods ranging from 45 to 240 min.

**Table 1:** Characteristics of the studies included in the systematic review and meta-analysis

Study	Subjects	*n*	Study design	Physical exercise	Blood draw	Measurement methods
Bouillon (2006) [22]	Seven female triathletes and seven recreational active females, 32.8 ± 4.5 years	14	RCT, cross-over design (exercise, control)	Cycling, 60 min at 60% *V*O_2max_ (moderate)	Pre, post, recovery (120 min)	Cytotoxicity in %; whole blood, 51Cr release assay, K562 cells; NKCA, NK-cell count (via flow cytometry)
Brenner (1996) [11]	Moderately fit males, 27.1 ± 3.0 years	11	RCT, cross-over design (23 °C-exercise, 23 °C-control, 40 °C-exercise, 40 °C-control)	Cycling, 2 × 30 min at 50% *V*O_2max_ (moderate)	Pre, post, recovery (180 min)	Cytotoxicity in %; PBMC; 51Cr release assay, K562 cells; NKCA, NK-cell count (via flow cytometry)
Brenner (1999) [26]	Moderately fit males, 24.9 ± 2.3 years	8	RCT, cross-over-design (all-out, endurance, resistance, control)	Cycling/resistance training, 5 min at 90% (high), 20 min at 60% *V*O_2max_ (moderate), 5 strength exercises at 60% of 1-RM (moderate)	Pre, post, recovery (45 min)	Cytotoxicity in %; PBMC, 51Cr release assay, K562 cells; NKCA, NK-cell count (via flow cytometry)
Flynn (1999) [23]	Healthy females, 72.8 ± 4.2 years	29	RCT, exercise (*n* = 15) vs. control (*n* = 14)	Resistance training, 4 strength exercises at 80% of 1-RM (high)	Pre, post, recovery (120 min)	Cytotoxicity in %; whole blood, 51Cr release assay, K562 cells; NKCA, NK-cell count (via flow cytometry)
Gannon (1998) [12]	Recreational active males, 26.3 ± 5.4 years	10	RCT, cross-over design (exercise, naltrexone, control)	Cycling, 120 min at 65% *V*O_2max_ (vigorous)	Pre, post, recovery (120 min)	Cytotoxicity in %; PBMC, flow cytometric assay, K562 cells; NKCA, NK-cell count (via flow cytometry)
Lee (2005) [27]	Healthy males, 26.5 ± 1.5 years	18	RCT, exercise (*n* = 9) vs. control (*n* = 9)	Qi-training, 60 min exercise (light)	Pre, post, recovery (120 min)	Cytotoxicity in %; PBMC, LDH release of K562 cells; NKCA, NK-cell count (via flow cytometry)
McFarlin (2003) [28]	Healthy males, 21.6 ± .4 years	10	RCT, cross-over design (two bouts, AM-only, PM-only, control)	Cycling, 3 × 20 min at 70% *V*O_2max_ with each segment initially 5 min at 50% *V*O_2max_ (vigorous)	Pre, post, recovery (120 min)	Cytotoxicity in %; whole blood, 51Cr release assay, K562 cells; NKCA
McFarlin (2005) [24]	Postmenopausal females, 72.1 ± 6.4 years	25	CT, exercise (*n* = 19) vs. control (*n* = 6)	Resistance exercise, 9 strength exercises at 80% of 1-RM (high)	Pre, post, recovery (120 min)	Cytotoxicity in %; whole blood, 51Cr release assay; K562 cells; NKCA, NK-cell count (via flow cytometry)
Miles (2002) [29]	Male runners, 31.2 ± 7.1 years	10	RCT, exercise (*n* = 6) vs. control (*n* = 4)	Running, 60 min treadmill at 80% *V*O_2max_ (vigorous)	Pre, post, recovery (90 min)	Lytic index; whole blood, 51Cr release assay, K562 cells; NKCA, NK-cell count (via flow cytometry)
Moyna (1996) [31]	Healthy males and females, 24.1 ± 2.8 years	64	RCT, exercise (*n* = 32) vs. control (*n* = 32)	Cycling, 18 min in total with 6 min at 55% *V*O_2peak_, 6 min at 70%, 6 min at 85% (vigorous)	Pre, post, recovery (120 min)	Lytic units; whole blood, 51Cr release assay, K562 cells; NKCA, NK-cell count (via flow cytometry)
Palmo (1995) [30]	Non-trained males, 20–29 years	16	CT, exercise (*n* = 8) vs. control (*n* = 8)	Eccentric exercise, 4 × 5 min one-legged exercise (moderate)	Pre, post, recovery (240 min)	Cytotoxicity in %; PBMC, 51Cr release assay; K562 cells; NKCA
Strasner (1997) [25]	Healthy females, 24.8 ± 3.9 years	8	RCT, cross-over design (vigorous, light, control)	Cycling, 25 min at 80% *V*O_2max_ (vigorous), 25 min at 40% *V*O_2max_ (light)	Pre, post, recovery (90 min)	Cytotoxicity in %; PBMC, 51Cr release assay, K562 cells; NKCA

In ten studies [11, 12, 22–28, 30], NKCA was expressed as cytotoxicity in percent (i.e., percentage of target cell lysis) and in two studies NKCA was reported in form of Lytic Units (i.e., number of effector cells required to lyse a certain percentage of target cells) [29, 31]. In half of the 12 studies, whole blood samples were mixed with the target cells (i.e., K562 leukaemia cells) [22–24, 28, 29, 31], while the other half used peripheral blood mononuclear cells (PBMC) [11, 12, 25–27, 30]. The Chromium-51 (Cr^51^) release assay was applied to quantify cytotoxicity in 11 studies [11, 12, 22–26, 28–31], while one study tested for lactate dehydrogenase (LDH) release of the target cells [27]. Moreover, nine studies reported absolute numbers of NK-cell count in response to exercise [11, 12, 22–24, 26, 27, 29, 31]. A summary of the basic characteristics of each study is given in Table 1.

### Meta-analysis of exercise effects

**Fig. 2:**
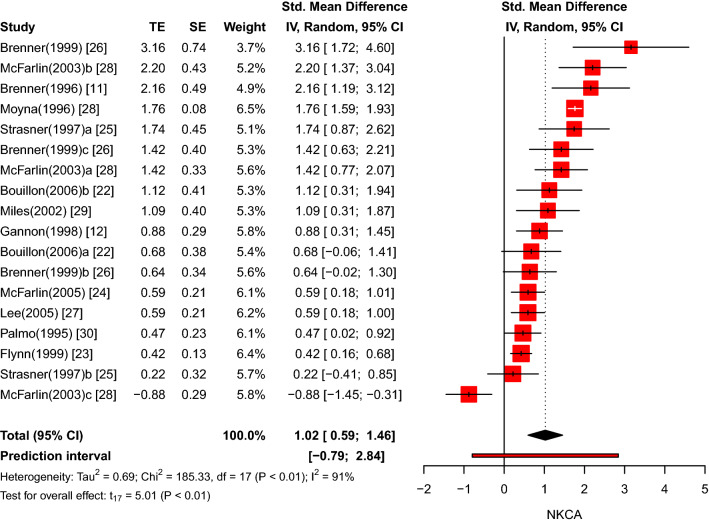
Forest plot of NKCA results (exercise versus control group) immediately after physical exercise (pre versus post)

Overall, 12 studies reporting 18 effect sizes were available for the quantitative synthesis. There was a total of 223 participants included in the analysis. The overall Hedges' *g* showed a large effect size (*k* = 18, *g* = 1.02, 95% CI 0.59–1.46, p < 0.01) with large heterogeneity (*τ*^2^ = 0.69, *p* < 0.01, *I*^2^ = 91%). The prediction interval ranged from *g* = − 0.79 to 2.84, meaning that the effect size can substantially vary across settings (Fig. 2).

**Fig. 3:**
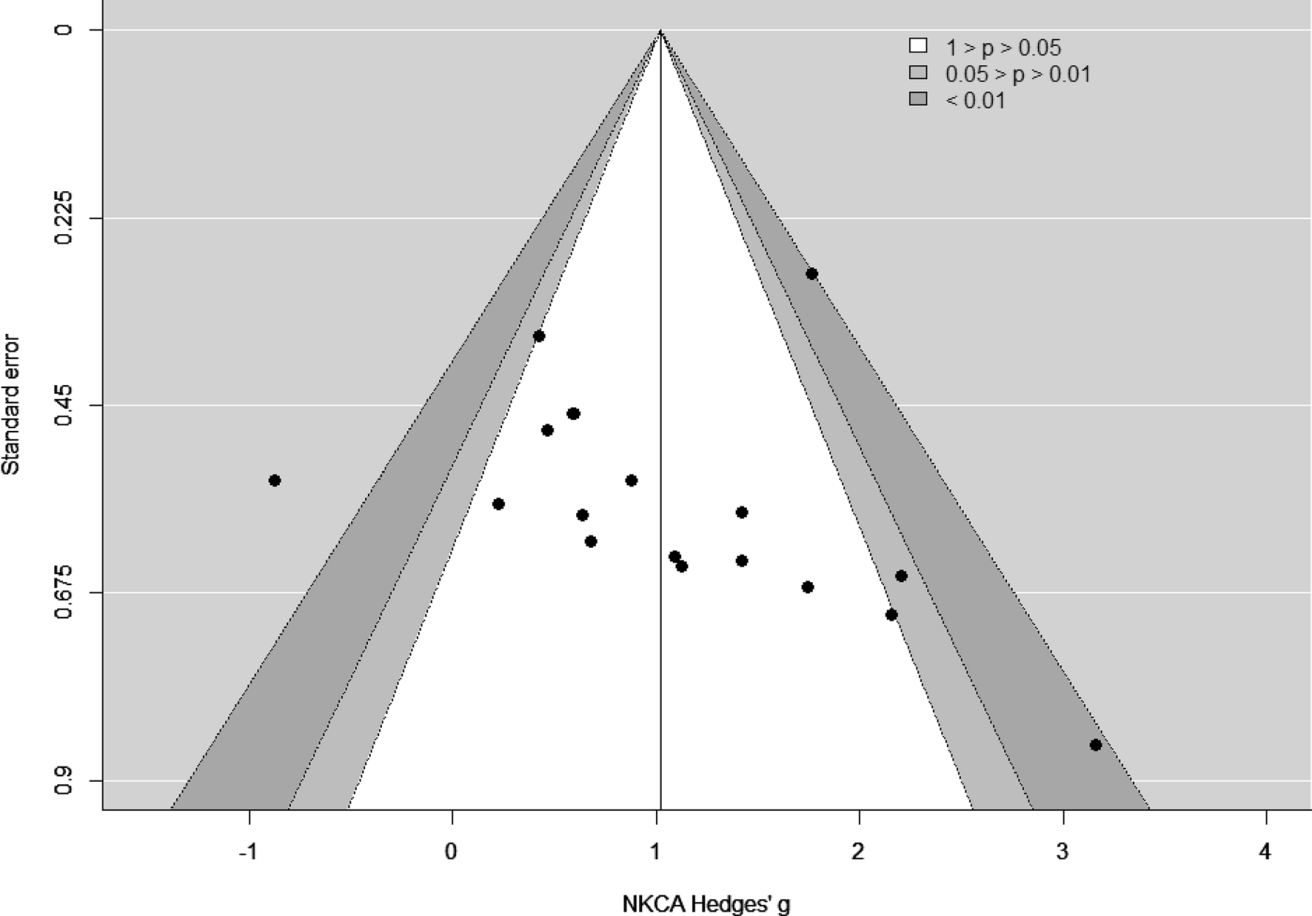
Funnel plot of NKCA results (exercise versus control group) immediately after physical exercise (pre versus post)

The visual inspection of the funnel plot suggested asymmetry in the data (Fig. 3). However, this asymmetry was not statistically significant according to Egger's test (slope = 1.35, one-tailed *p* = 0.38). Nevertheless, after sensitivity analysis two outliers were identified with Cook’s Distance larger than 0.45 [20], hence the Moyna study [31] and one out of three effect sizes from the McFarlin study [28] were withdrawn from further analyses.

Rerunning the meta-analysis without outliers revealed a slightly increased effect size (*k* = 16, *g* = 1.08, 95% CI 0.69–1.47, p < 0.01) and a narrower prediction interval of *g* = − 0.49 to 2.64 (see ESM Figure A) due to lower between-study heterogeneity (*τ*^2^ = 0.50, *p* < 0.01, *I*^2^ = 73%). To further corroborate the results, changes in the experimental and passive control groups were evaluated separately. The effect of physical exercise on NKCA was large and significant in the experimental group (*k* = 16, *g* = 1.59, 95% CI 1.14–2.05, *p* < 0.01), whereas no significant deviation was detected in the control groups (*k* = 16, *g* = 0.10, 95% CI − 0.01 to 0.21, *p* = 0.06), confirming that both conditions worked as expected. The forest plots are available in ESM Figure B and Figure C, respectively.

### Moderator Analyses

To better explain the large between-study heterogeneity, a moderator analysis was conducted. Individual-level moderators (i.e., gender, age), exercise-level moderators (i.e., type, intensity), and a method-level moderator (i.e., type of blood sample) were assessed by subgroups analyses, while meta-regression was applied to examine a confounding effect of NK-cell count.

The effect size was stronger in males (*k* = 10, *g* = 1.29, 95% CI 0.72–1.87) than in females (*k* = 6, *g* = 0.72, 95% CI 0.18–1.25), however, this difference was not significant (*χ*^2^ = 3.08, *df* = 1, *p* = 0.08). The comparison of young and old subgroups revealed a significant between-subgroup difference (*χ*^2^ = 10.69, *df* = 1, *p* < 0.01) with a large effect size among young participants (*k* = 14, *g* = 1.18, 95% CI 0.74–1.61) and a smaller effect size among old participants (*k* = 2, *g* = 0.47, 95% CI − 0.52 to 1.46). However, this result needs to be considered in caution given that the absolute majority of studies had tested young participants.

On the exercise level, a significant difference (*χ*^2^ = 12.92, *df* = 1, p < 0.01) was detected between studies using resistance (*k* = 4, *g* = 0.48, 95% CI 0.33–0.63) compared to endurance exercise (*k* = 12, *g* = 1.30, 95% CI 0.81–1.78). Moreover, the intensity of exercise played a significant role (*χ*^2^ = 11.21, *df* = 3, *p* < 0.01) in the sense that high (*k* = 3, *g* = 1.27, 95% CI − 2.39 to 4.93) and vigorous intensity (*k* = 5, *g* = 1.41, 95% CI 0.76–2.06) led to larger effect sizes than moderate (*k* = 6, *g* = 1.01, 95% CI 0.37–1.65) and light exercise intensity (*k* = 2, *g* = 0.48, 95% CI − 1.68 to 2.64).

On the methodological level, no significant difference (*χ*^2^ = 0.14, *df* = 1, *p* = 0.71) was found for the type of blood sample used in the NKCA assay with whole blood (*k* = 7, *g* = 1.01, 95% CI 0.45–1.56) compared to PBMC (*k* = 9, *g* = 1.14, 95% CI 0.46–1.83).

Finally, it was tested if the effect of physical exercise on NKCA covaried with the number of NK-cells in the peripheral blood. Based on 9 studies reporting 11 effect sizes of NK-cell count along with NKCA values, a meta-regression was applied which did not reveal a significant confounding effect (*k* = 11, beta = 0.123,* R*^2^ = 0%, *p* = 0.55). Thus, NK-cell count could not explain the between-study heterogeneity. The relationship between the effect size and the NK-cell count is displayed on the bubble plot in ESM Figure D.

### Meta-analysis of recovery effects

**Fig. 4:**
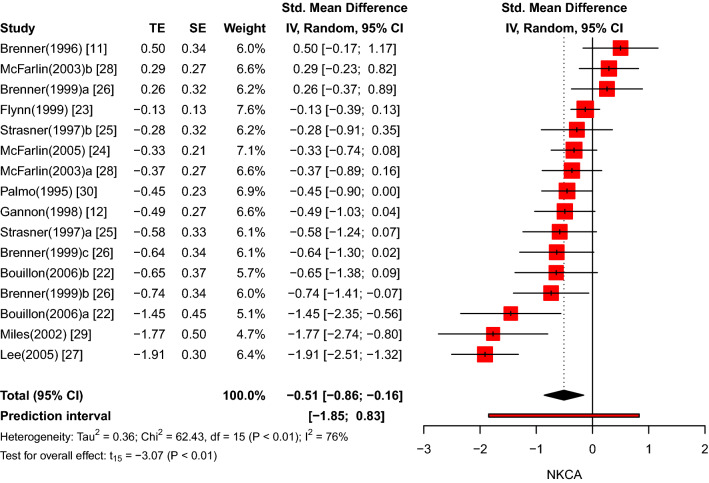
Forest plot of NKCA results (exercise versus control group) after recovery period (pre versus recovery)

Next, the effect of physical exercise on NKCA after a recovery period was examined. By comparing the pre-exercise NKCA values with the recovery NKCA values in both the exercise and control groups a moderate negative effect size was discovered (*k* = 16, *g* = − 0.51, 95% CI − 0.86 to − 0.16, *p* < 0.01).The prediction interval ranged from a huge negative to a large positive effect size (*g* = − 1.85 to 0.83), meaning that the effect size can still substantially vary across settings. As shown in Fig. 4, large between-study heterogeneity (*τ*^2^ = 0.36, *p* < 0.01, *I*^2^ = 76%) became evident.

**Fig. 5:**
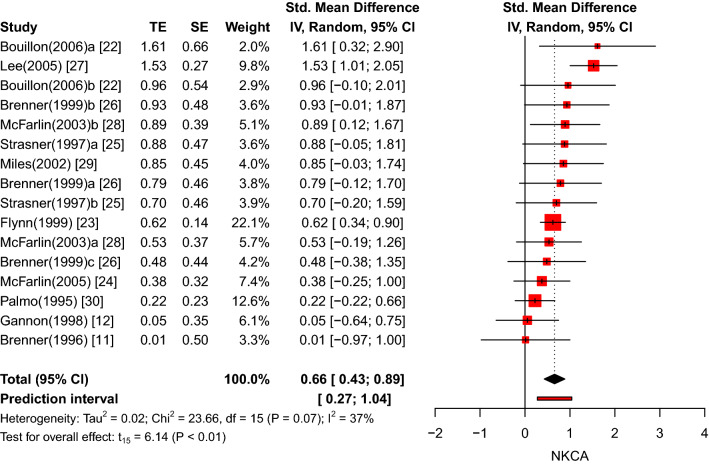
Forest plot of NKCA results in the control groups after recovery period (pre versus recovery)

Finally, the exercise and control groups were analysed separately. Within the exercise groups, no evidence was found for NKCA alterations subsequent to the recovery period (*k* = 16, *g* = 0.06, 95% CI − 0.37 to 0.50, *p* = 0.76; see ESM Figure E). In the control groups, however, the effect size was significant and moderately positive (*k* = 16, *g* = 0.66, 95% CI 0.43–0.89, *p* < 0.01), while the between-study heterogeneity was small and insignificant (*τ*^2^ = 0.02, *I*^2^ = 37%, *p* = 0.07). In other words, physical exercise did not significantly decrease the NKCA after the recovery period in the experimental groups, but the relative comparison with the elevated NKCA levels in the control groups suggested a negative overall effect. Figure 5 presents the forest plot for the control groups.

It can be assumed that the effect on NKCA was altered by the recovery period which varied considerably in length between the studies. Therefore, meta-regression models were run to analyse the impact of the recovery time in the overall sample (*k* = 16, beta = 0.00, *R*^2^ = 0%, *p* = 0.898), exercise groups only (*k* = 16, beta = 0.001, *R*^2^ = 0%, *p* = 0.810), and control groups only (*k* = 16, beta = 0.002, *R*^2^ = 0%, *p* = 0.372). All three meta-regression models turned out to be insignificant, that is, the duration of the recovery period did not impact the NKCA values, and thus, could not explain between-study heterogeneity.

## Discussion

This review contributes to the literature as it (a) provides systematic evidence for the positive effect of acute exercise on NKCA, (2) sheds more light on the moderating factors, and (3) contradicts with the widespread assumption of NKCA suppression in the hours after the exercise. These findings are important as they may contribute to evidence-based strategies for the prevention and treatment of virus infections and cancer.

The meta-analysis provides solid evidence for elevated NKCA through performing sports activities which was previously suggested by Zimmer et al. on basis of a systematic review [5]. A large impact on NKCA becomes evident for the comparison of the exercise groups with the control groups, and for the separate analysis of the exercise groups. There is a large dispersion of effect sizes between the studies which can be partly explained by individual factors, such as sex and age, and exercise-related factors, such as type and intensity. On the contrary, the biomaterial used in the assay (whole blood, PBMC) and the mobilization of NK-cells into the peripheral blood do not significantly explain heterogeneity.

In regards of the individual factors this study potentially suggests a trend towards males being more sensitive to physical exercise in terms of NKCA elevation. So far, investigations on the effect of gender on NKCA are missing. As stated by Klein and Flanagan [33], differences between both genders exist (e.g., higher NK-cell numbers in males) and hormonal differences could be an explanation. Furthermore, age is indicated as an individual factor affecting NKCA. Positive effects of exercise seem to be more evident among younger people which could be explained by a decreased immune response in aged individuals, so called immunosenescence [34], However, more systematic studies are needed to draw meaningful conclusions since most experiments to date focus on younger adults.

Besides individual factors, clearer implications can be drawn regarding exercise-related factors. Endurance training boosts the NKCA level more effectively than resistance training. This is in line with previous results showing stronger alterations by endurance exercise [35–37]. Furthermore, and in line with a previous meta-analysis [7], more intense forms of exercise cause a higher impact on NKCA.

From a methodological perspective, the different blood sampling approaches used in NKCA research have been assumed to jeopardize the comparability of study results [5], As outlined in this systematic review, cytotoxicity assays have been either performed by mixing whole blood samples or PBMC with the target cells. While whole blood sample approaches might reflect the in vivo situation more precisely, PBMC approaches might better control for cytokines and hormones which also influence the integrity of target cells. The subgroup analysis finds no significant difference between the use of whole blood and PBMC approaches, thus the results reduce the widespread methodological concern. Nevertheless, combining different biomaterial such as whole blood, PBMCs or isolated NK-cells, and different measurement methods such as Cr^51^ release assay or flow cytometry-based measurements, would provide more understanding about the influence of methodological aspects. Particularly with regards to the use of radioactive material in the Cr^51^ release assay, it is necessary to identify reliable methods without radioactive or toxic substances for a safe measurement of cytotoxicity.

Another critical issue discussed in the literature relates to the confounding effect of NK-cell mobilization. Since NK-cell counts vary significantly in response to physical activity, it has been assumed that the sport induced elevation of NKCA might simply be due to an absolute and/or proportional increase in NK-cell numbers [7]. The meta-analytical results provide counterevidence to this assumption. Hence, it should be proceeded from the assumption that the functional change in NKCA exists independently from the quantitative change in NK-cell number. However, this interesting finding needs more research attention, particularly with regards to the distribution of NK-cell subsets which were not considered in this analysis due to a lack of more sophisticated raw data. More precisely, CD56^dim^ NK-cells have a different cytolytic capacity compared to CD56^bright^ NK-cells, and thus, a changing proportion could still be associated with an alteration in NKCA. Unfortunately, most of the included studies did not provide information of changes in NK-cell subsets. Therefore, it remains unclear whether or not changes in NKCA could be due to changes in NK-cell subsets.

A previous review has reported negative changes in NKCA in the hours after performing sports which can be prolonged for up to 24 h [7]. This is relevant as a decreased immune function provides an ‘open window’ for opportunistic infections. Interestingly, this meta-analysis provides counterevidence to this assumption. In the exercise groups, the elevated NKCA values return to baseline during the first 1–2 h of recovery, but not below the pre-exercise values. In the control groups, however, a significant gain in NKCA becomes evident. This unexpected finding raises questions about the blood sample management during the experiments. Possibly, the NKCA assays were not conducted immediately after the blood draw, and thus, biochemical processes might have taken place in the meantime.

It remains speculative why NKCA was elevated in the control groups over the course of the experiments. More importantly, this finding provides an explanation for the prevailing opinion that NKCA is suppressed in the hours after the exercise. This conclusion seems to be based on a statistical artefact since the control groups serve as a baseline measure and the NKCA outcomes shift for some unknown reasons. Systematic research is needed to investigate this phenomenon.

The results of the presented meta-analysis should be considered under its limitations. First, only 12 studies were included in the meta-analysis due to the inclusion criteria providing a meaningful data base. Nevertheless, these studies are still heterogeneous regarding their exercise regimens and the measurement of NKCA. These differences were systematically addressed in the meta-analysis, however, in some cases skewed data (e.g., age subgroup analysis) produced underpowered results. Noteworthy, the most recent study meeting the inclusion criteria was published in 2006. Since then, several studies addressed quite specific research questions such as the role of carbohydrate consumption [38], latent virus infection [39] or hypoxia [40] in the context of acute physical exercise and NKCA. Given the essential literature gaps outlined above, this study could once again inspire more fundamental research hypotheses in the field of exercise immunology and sports medicine.

## Conclusion

In conclusion, this work represents an important next step towards a deeper understanding of NKCA in response to physical exercise by identifying the effect size across studies and controlling for the confounding effect of NK-cell mobilization. Whereas the study provides a more coherent picture of the impact of sports on NKCA, future investigations will be required to better explain the moderating factors in play. Of particular importance will be research explaining interindividual heterogeneity as a basis for the development of tailored exercise strategies to supplement with personalized medicine. In addition, the effect of sports on NKCA should be studied in patients suffering from specific cancer types to analyse in how far physical exercise can improve the outcome of immunotherapies.

## Supplementary Information

Below is the link to the electronic supplementary material.

Supplementary file1 (PDF 226 KB)

## Declarations

### Availability of data and material

Data are available on request from the authors.
