# Supplementary material for: The Effect of Acute Physical Exercise on NK-Cell Cytolytic Activity: A Systematic Review and Meta-Analysis
Source: Sports Med. 2021 Jan 4;51(3):519–30. doi: 10.1007/s40279-020-01402-9 (PMC7900082; doi:10.1007/s40279-020-01402-9)
Supplement: Supplementary file 1 — Supplementary file1 (PDF 226 KB) [file 40279_2020_1402_MOESM1_ESM.pdf]

Article title: The effect of acute physical exercise on NK-cell cytolytic activity: A systematic review and meta-analysis
Journal: Sports Medicine
Authors: Christopher Rumpf, Sebastian Proschinger, Alexander Schenk, Wilhelm Bloch, Amit Lampit, Florian Javelle, and Philipp Zimmer
Contact: Philipp Zimmer, Institute of Sport and Sport Science, Technical University Dortmund, Germany, philipp.zimmer@tu-dortmund.de

Electronic Supplementary Material (ESM)

ESM Table A. PEDro scale

Study	Eligibility criteria	Random allocation	Concealed allocation	Similar at baseline	Subjects blinded	Therapists blinded	Assessors blinded	>85% follow-up	intention-to-treat analysis	Between-group comparisons	Point and variability measures	Total PEDro score
Bouillon (2006) [22]	1	1	0	1	n/a	n/a	0	1	1	1	1	6
Flynn (1999) [23]	1	1	0	1	n/a	n/a	0	1	1	1	1	6
Gannon (1998) [12]	1	1	0	1	n/a	n/a	0	1	1	1	1	6
Lee (2005) [27]	1	1	0	1	n/a	n/a	0	1	1	1	1	6
Moyna (1996) [31]	1	1	0	1	n/a	n/a	0	1	1	1	1	6
Strasner (1997) [25]	1	1	0	1	n/a	n/a	0	1	1	1	1	6
McFarlin (2003) [28]	1	0	0	1	n/a	n/a	0	1	1	1	1	5
Palmo (1995) [30]	1	0	0	1	n/a	n/a	0	1	1	1	1	5
Brenner (1999) [26]	1	1	0	1	n/a	n/a	0	1	1	1	1	6
Brenner (1996) [11]	1	1	0	1	n/a	n/a	0	1	1	1	1	6
McFarlin (2003) [28]	1	1	0	1	n/a	n/a	0	1	1	1	1	6
Miles (2002) [29]	1	1	1	1	n/a	n/a	0	1	1	1	1	7

Criteria (2-11) were used to calculate the total PEDro score. Each criterion was scored as either 1 or 0 according to whether the criteria was met or not. n/a = not applicable

Study	TE	SE	Weight	Std. Mean Difference IV, Random, 95% CI
Brenner(1999)a [26]	3.16	0.74	3.8%	3.16 [1.72; 4.60]
McFarlin(2003)b [28]	2.20	0.43	5.8%	2.20 [1.37; 3.04]
Brenner(1996) [11]	2.16	0.49	5.3%	2.16 [1.19; 3.12]
Strasner(1997)a [25]	1.74	0.45	5.6%	1.74 [0.87; 2.62]
Brenner(1999)c [26]	1.42	0.40	6.0%	1.42 [0.63; 2.21]
McFarlin(2003)a [28]	1.42	0.33	6.5%	1.42 [0.77; 2.07]
Bouillon(2006)b [22]	1.12	0.41	5.9%	1.12 [0.31; 1.94]
Miles(2002) [29]	1.09	0.40	6.0%	1.09 [0.31; 1.87]
Gannon(1998) [12]	0.88	0.29	6.7%	0.88 [0.31; 1.45]
Bouillon(2006)a [22]	0.68	0.38	6.2%	0.68 [−0.06; 1.41]
Brenner(1999)b [26]	0.64	0.34	6.4%	0.64 [−0.02; 1.30]
McFarlin(2005) [24]	0.59	0.21	7.3%	0.59 [0.18; 1.01]
Lee(2005) [27]	0.59	0.21	7.3%	0.59 [0.18; 1.00]
Palmo(1995) [30]	0.47	0.23	7.1%	0.47 [0.02; 0.92]
Flynn(1999) [23]	0.42	0.13	7.6%	0.42 [0.16; 0.68]
Strasner(1997)b [25]	0.22	0.32	6.5%	0.22 [−0.41; 0.85]
**Total (95% CI)**			**100.0%**	**1.08 [0.69; 1.47]**
**Prediction interval**				**[−0.49; 2.64]**

Heterogeneity: $Tau^2 = 0.50$; $Chi^2 = 55.49$, df = 15 ($P < 0.01$); $I^2 = 73\%$

Test for overall effect: $t_{15} = 5.86$ ($P < 0.01$)

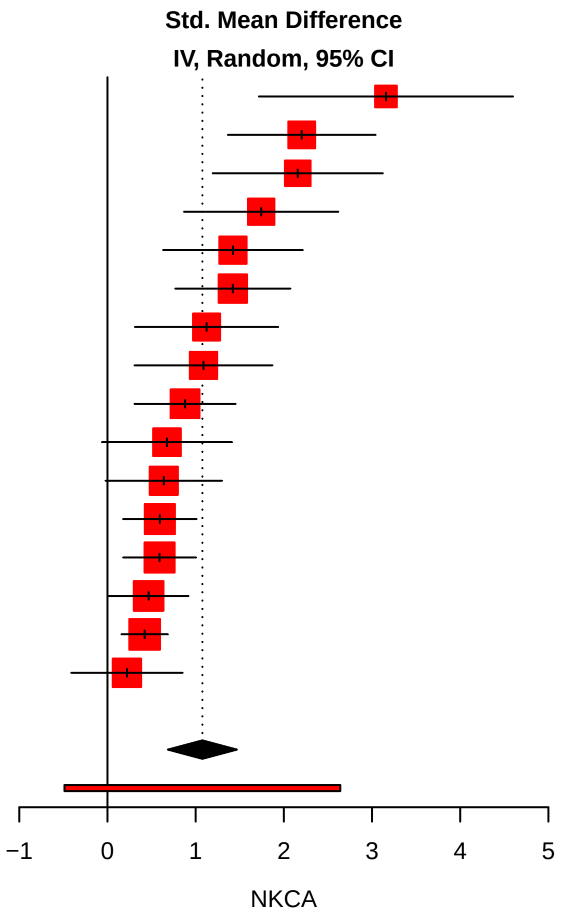

ESM Figure A. Forest plot of NKCA results (exercise versus control group) immediately after physical exercise (pre versus post) without outliers

Study	TE	SE	Weight	Std. Mean Difference IV, Random, 95% CI
Brenner(1999)a [26]	3.82	0.93	3.4%	3.82 [2.00; 5.63]
Lee(2005) [27]	2.78	0.42	6.3%	2.78 [1.96; 3.59]
McFarlin(2003)b [28]	2.68	0.50	5.7%	2.68 [1.69; 3.66]
Strasner(1997)a [25]	2.30	0.54	5.5%	2.30 [1.24; 3.36]
Brenner(1999)c [26]	2.25	0.53	5.6%	2.25 [1.21; 3.29]
Brenner(1996) [11]	2.17	0.49	5.8%	2.17 [1.20; 3.13]
Miles(2002) [29]	1.70	0.48	5.9%	1.70 [0.75; 2.65]
Bouillon(2006)a [22]	1.69	0.49	5.8%	1.69 [0.73; 2.65]
Bouillon(2006)b [22]	1.68	0.49	5.8%	1.68 [0.72; 2.63]
Gannon(1998) [12]	1.45	0.34	6.8%	1.45 [0.79; 2.11]
McFarlin(2003)a [28]	1.23	0.32	7.0%	1.23 [0.61; 1.85]
Brenner(1999)b [26]	1.02	0.36	6.7%	1.02 [0.30; 1.73]
Palmo(1995) [30]	0.95	0.25	7.3%	0.95 [0.46; 1.44]
Flynn(1999) [23]	0.91	0.14	7.8%	0.91 [0.62; 1.19]
McFarlin(2005) [24]	0.46	0.21	7.6%	0.46 [0.05; 0.86]
Strasner(1997)b [25]	0.44	0.33	6.9%	0.44 [−0.20; 1.09]
**Total (95% CI)**			**100.0%**	**1.59 [1.14; 2.05]**
**Prediction interval**				**[−0.16; 3.35]**

Heterogeneity: $Tau^2 = 0.62$; $Chi^2 = 69.56$, df = 15 ($P < 0.01$); $I^2 = 78\%$

Test for overall effect: $t_{15} = 7.44$ ($P < 0.01$)

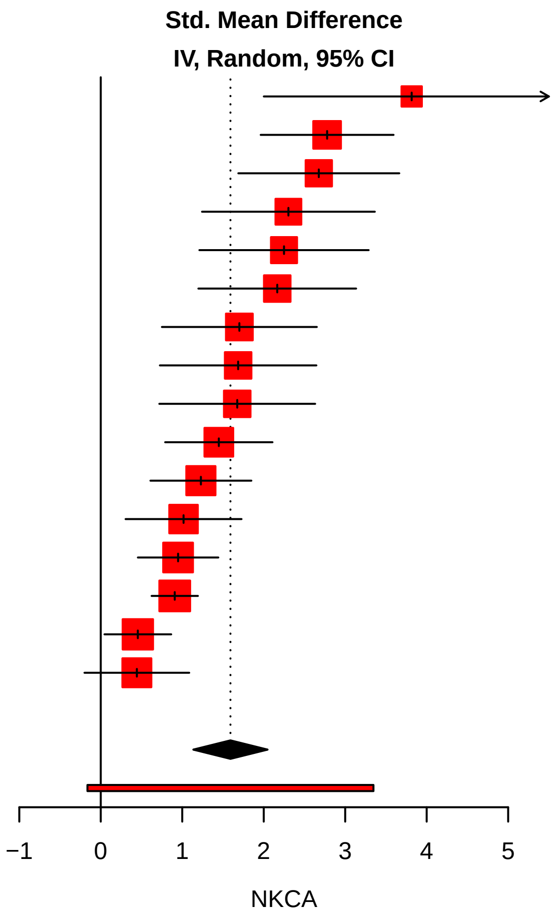

ESM Figure B. Forest plot of NKCA results in the exercise groups immediately after physical exercise (pre versus post)

Study	TE	SE	Weight	Std. Mean Difference IV, Random, 95% CI
Brenner(1999)c [26]	0.54	0.33	3.7%	0.54 [−0.11; 1.19]
Bouillon(2006)a [22]	0.40	0.36	3.1%	0.40 [−0.31; 1.11]
Lee(2005) [27]	0.33	0.20	9.6%	0.33 [−0.07; 0.73]
Bouillon(2006)b [22]	0.29	0.36	3.1%	0.29 [−0.42; 0.99]
Strasner(1997)a [25]	0.24	0.32	3.9%	0.24 [−0.39; 0.87]
Strasner(1997)b [25]	0.24	0.32	3.9%	0.24 [−0.39; 0.87]
Brenner(1999)b [26]	0.22	0.32	3.9%	0.22 [−0.41; 0.86]
Palmo(1995) [30]	0.16	0.22	8.0%	0.16 [−0.28; 0.60]
Brenner(1999)a [26]	0.13	0.32	3.9%	0.13 [−0.50; 0.76]
Flynn(1999) [23]	0.10	0.13	23.5%	0.10 [−0.15; 0.36]
Gannon(1998) [12]	0.08	0.27	5.7%	0.08 [−0.44; 0.61]
Brenner(1996) [11]	−0.02	0.33	3.6%	−0.02 [−0.67; 0.63]
Miles(2002) [29]	−0.09	0.34	3.5%	−0.09 [−0.76; 0.58]
McFarlin(2005) [24]	−0.14	0.21	9.5%	−0.14 [−0.54; 0.26]
McFarlin(2003)a [28]	−0.24	0.27	5.6%	−0.24 [−0.76; 0.28]
McFarlin(2003)b [28]	−0.24	0.27	5.6%	−0.24 [−0.76; 0.28]
**Total (95% CI)**			**100.0%**	**0.10 [−0.01; 0.21]**
**Prediction interval**				**[−0.01; 0.21]**

Heterogeneity: $Tau^2 = 0$; $Chi^2 = 9.58$, df = 15 (P = 0.85); $I^2 = 0\%$

Test for overall effect: $t_{15} = 2.02$ (P = 0.06)

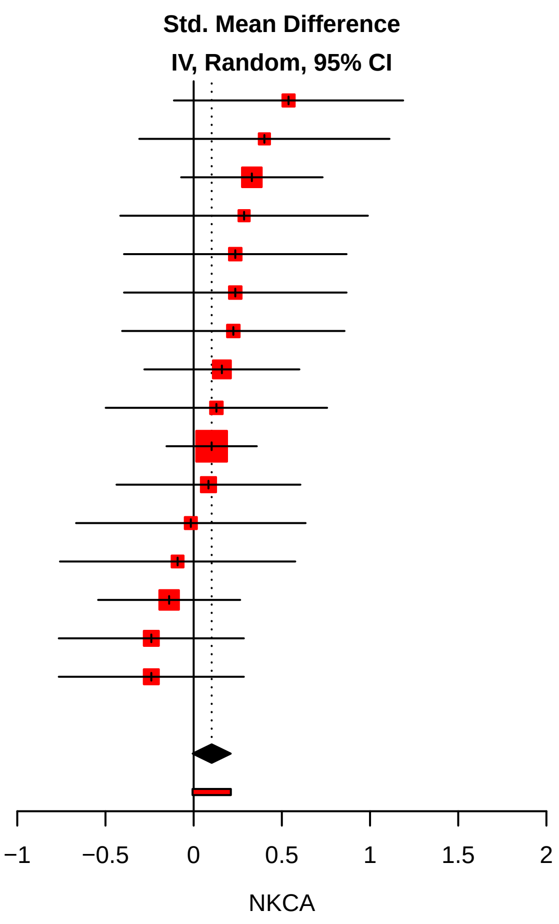

ESM Figure C. Forest plot of NKCA results in the control groups immediately after physical exercise (pre versus post)

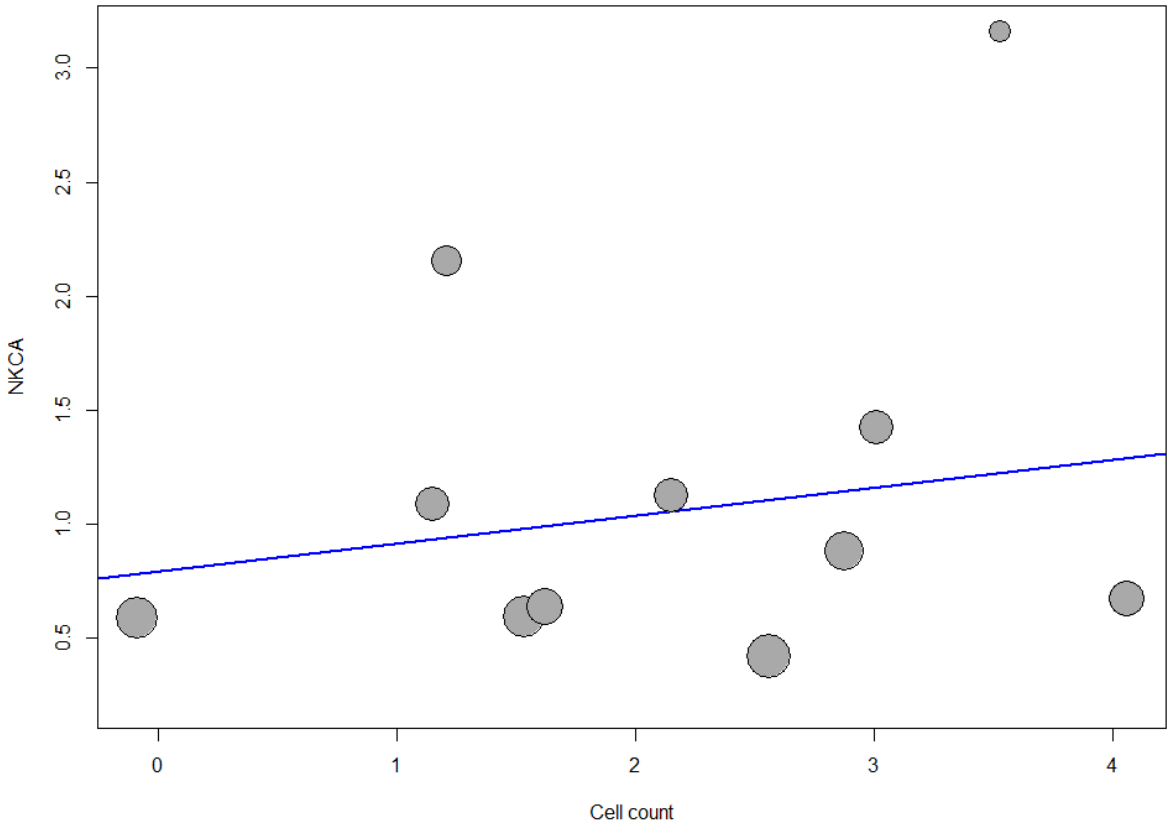

ESM Figure D. Bubble plot of meta-regression results immediately after physical exercise (pre versus post)

Study	TE	SE	Weight	Std. Mean Difference IV, Random, 95% CI
McFarlin(2003)b [28]	1.16	0.18	6.3%	1.16 [0.81; 1.52]
Brenner(1999)a [26]	1.09	0.22	6.2%	1.09 [0.66; 1.53]
Flynn(1999) [23]	0.49	0.13	6.5%	0.49 [0.24; 0.75]
Brenner(1996) [11]	0.49	0.16	6.4%	0.49 [0.17; 0.80]
Strasner(1997)b [25]	0.45	0.22	6.2%	0.45 [0.02; 0.88]
Strasner(1997)a [25]	0.35	0.22	6.2%	0.35 [−0.08; 0.77]
Bouillon(2006)b [22]	0.22	0.24	6.1%	0.22 [−0.25; 0.68]
McFarlin(2003)a [28]	0.22	0.18	6.3%	0.22 [−0.13; 0.56]
Bouillon(2006)a [22]	0.11	0.24	6.1%	0.11 [−0.35; 0.57]
McFarlin(2005) [24]	0.08	0.10	6.6%	0.08 [−0.11; 0.28]
Brenner(1999)b [26]	0.08	0.21	6.2%	0.08 [−0.34; 0.49]
Brenner(1999)c [26]	−0.13	0.21	6.2%	−0.13 [−0.55; 0.29]
Palmo(1995) [30]	−0.22	0.22	6.2%	−0.22 [−0.66; 0.22]
Gannon(1998) [12]	−0.46	0.18	6.3%	−0.46 [−0.82; −0.11]
Lee(2005) [27]	−0.58	0.21	6.2%	−0.58 [−0.98; −0.17]
Miles(2002) [29]	−2.50	0.29	5.9%	−2.50 [−3.06; −1.93]
**Total (95% CI)**			**100.0%**	**0.06 [−0.37; 0.50]**
**Prediction interval**				**[−1.71; 1.84]**

Heterogeneity: $Tau^2 = 0.64$; $Chi^2 = 178.13$, df = 15 ($P < 0.01$); $I^2 = 92\%$

Test for overall effect: $t_{15} = 0.31$ ($P = 0.76$)

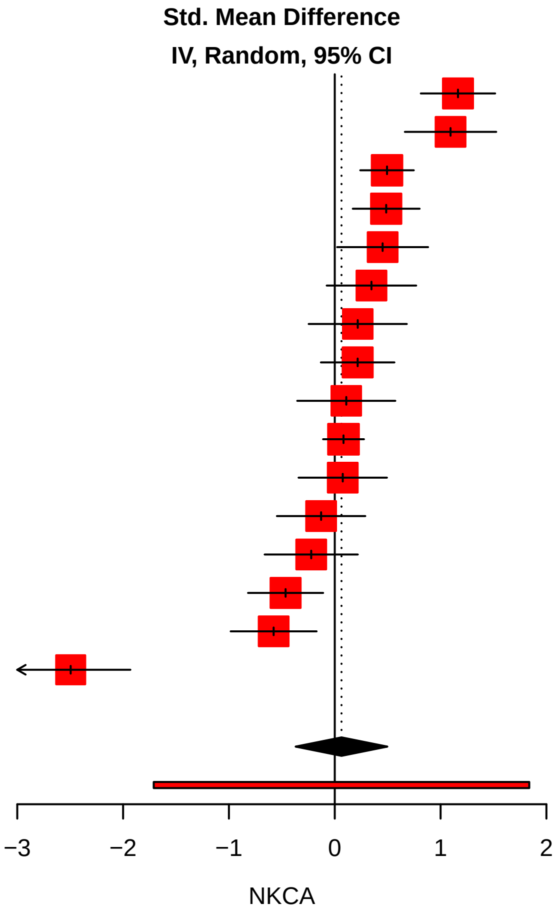

ESM Figure E. Forest plot of NKCA results in the exercise groups after the recovery period (pre versus recovery)
